# Optimizing personalized screening intervals for clinical biomarkers using extended joint models

**DOI:** 10.1080/02664763.2025.2505636

**Published:** 2025-05-30

**Authors:** Nobuhle Nokubonga Mchunu, Henry Mwambi, Tarylee Reddy, Nonhlanhla Yende-Zuma, Dimitris Rizopoulos

**Affiliations:** a Biostatistics Research Unit, South African Medical Research Council, Durban, South Africa; b School of Mathematics, Statistics and Computer Science, University of KwaZulu-Natal, Pietermaritzburg, South Africa; c Centre for the AIDS Programme of Research in South Africa (CAPRISA), Durban, South Africa; d Department of Biostatistics Erasmus University Medical Center, Rotterdam, The Netherlands; e Department of Epidemiology, Erasmus University Medical Center, Rotterdam, The Netherlands

**Keywords:** CD4 count, cross validation, multivariate joint models, personalized screening intervals, super learning, 92B15, 62H99, 62N01, 62P10

## Abstract

This research advances joint modeling and personalized scheduling for HIV and TB by incorporating censored longitudinal outcomes in multivariate joint models, providing a more flexible and accurate approach for complex data scenarios. Inspired by the SAPiT study, we deviate from standard model selection procedures by using super learning techniques to identify the optimal model for predicting future events in event-free subjects. Specifically, the Integrated Brier score and Expected Predictive Cross-Entropy (EPCE) identified the multivariate joint model with the parameterization of the area under the longitudinal profiles of CD4 count and viral load as optimal and strong predictors of death. Integrating this model with a risk-based screening strategy, we recommend extending intervals to 10.3 months for stable patients, with additional measurements every 12 months. For patients with deteriorating health, we suggest a 3.5-month interval, followed by 6.2 months, and then annual screenings. These findings refine patient care protocols and advance personalized medicine in HIV/TB co-infected individuals. Furthermore, our approach is adaptable, allowing adjustments based on patients' evolving health status. While focused on HIV/TB co-infection, this method has broader applicability, offering a promising avenue for biomarker studies across various disease populations and potential for future clinical trials and biomarker-guided therapies.

## 1. Introduction

The Human Immunodeficiency Virus (HIV) continues to be a serious global health threat, impacting millions of people globally and accounting for an estimated 37.7 million infections. In the year 2021 alone, HIV-related illnesses claimed the lives of 680,000 people [28]. In South Africa, the gravity of this challenge is evident, with an estimated overall national HIV prevalence of 14.0% (95% CI: 13.1–15.0) reported in the national HIV prevalence survey conducted in South Africa in 2017 [64]. HIV disease progression biomarkers, such as CD4 count and HIV-1 RNA viral load (or simply viral load), play a critical role in monitoring the disease course and guiding treatment decisions [34]. Despite significant progress in HIV treatment and the universal availability of antiretroviral therapy (ART), there persists a need to comprehensively understand the dynamic nature of disease progression and optimize treatment strategies for individual patients.

**Figure 1.**
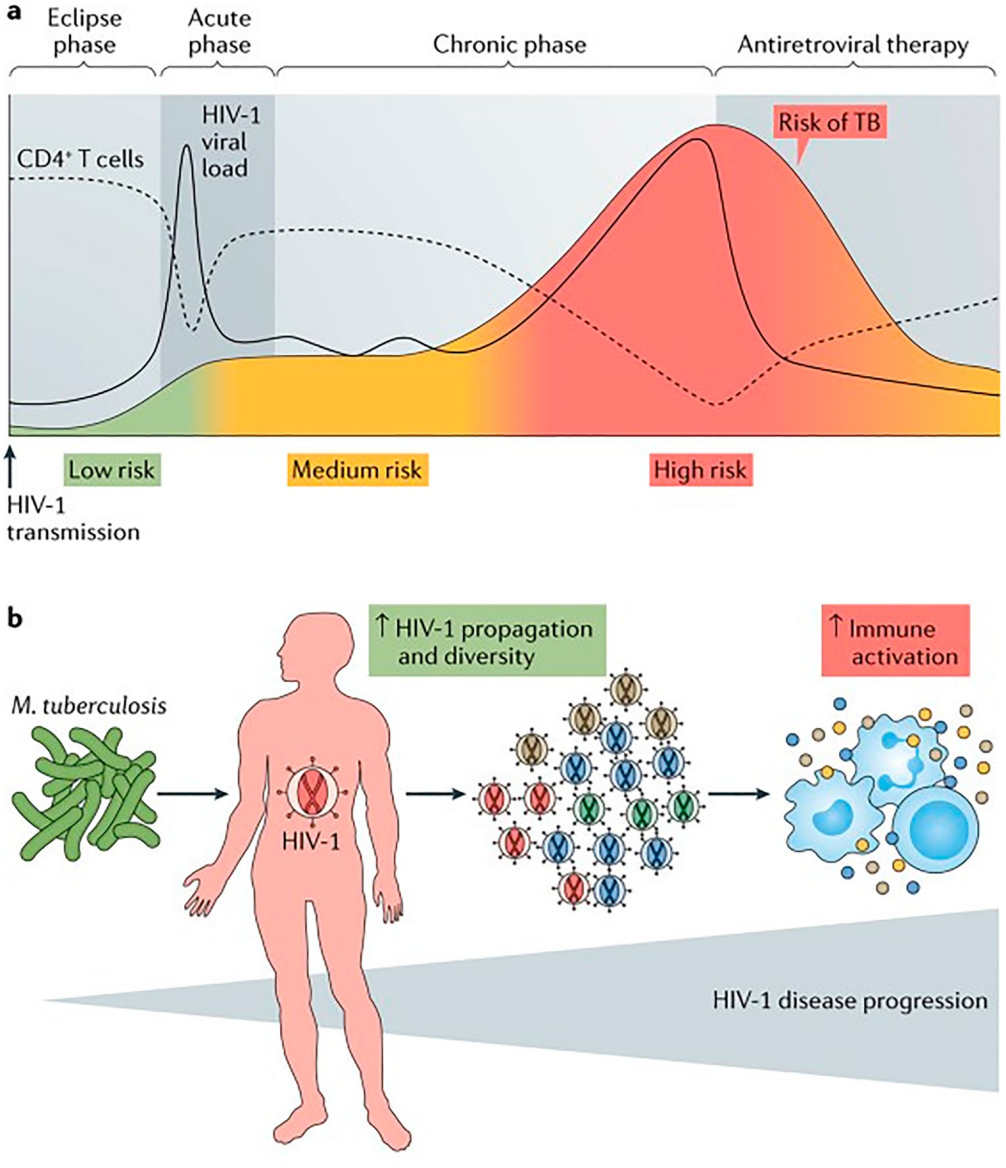
Illustration of the relationship between CD4 count and viral for TB/HIV co-infected individuals (**Source**: Bell *et al.*, 2018).

The routine monitoring of CD4 count and viral load as the two strongest correlates and surrogate markers of HIV disease progression is crucial in HIV management, allowing healthcare professionals to tailor treatment strategies to suit individual patient needs and optimize health outcomes [37]. However, the introduction of universal test and treat has put less emphasis on the importance CD4 count testing and the viral load is now widely preferred. Nonetheless, monitoring CD4 count is essential in HIV management [25] especially for people that present late to care as they are more susceptible to opportunistic infections such as Tuberculosis (TB) [18,50]. Figure 1 adapted from [6] illustrates the correlation between CD4 count and viral load for an untreated HIV infection with TB co-infection, where a declining CD4 count indicates a compromised immune system. Conversely, elevated viral load levels indicate active viral replication and an increased risk of disease progression [30].

Prior research has predominantly modeled CD4 count and viral load separately, overlooking their correlation despite both reflecting the progression of HIV infection [15,74]. In the era of personalized medicine, integrating these biomarkers into individualized screening intervals is crucial for optimizing HIV care [23]. This study bridges this gap by modeling CD4 count and viral load simultaneously, accounting for their interdependencies, and facilitating the optimization of follow-up schedules. By understanding their joint trajectory, healthcare providers can tailor monitoring strategies to match each patient's unique health profile, enabling more precise, efficient, and patient-centered HIV management. This approach has the potential to transform HIV care and advance personalized healthcare interventions. However, personalized screening strategies, particularly in TB/HIV co-infection, have been underexplored.

Statistical modeling techniques aimed at enhancing medical screening approaches have garnered significant interest in academic literature over the past two decades. The majority of these methodologies have primarily relied on Markov and multistate models, with a predominant focus on cancer-related data [3–5,7,44,45]. In particular, Markov decision processes (MDPs) and partially observable Markov decision processes (POMDPs) have been used to optimize screening schedules and treatment for chronic diseases [16,66]. The primary drawback of these methods lies on their inherent memoryless property, meaning that the future value of the longitudinal biomarker is determined solely by its current value. While this simplifies modeling and computations, it might not fully capture the complexities of disease progression, which may depend on historical information, treatment responses, or other long-term factors. In addition, these methods exploit population-level joint distributions of disease states which may overlook patient-specific characteristics that influence disease trajectories. Personalized medicine often requires considering individual variability.

Joint modeling [53,59,60,75] emerges as a powerful tool that addresses all the shortfalls of traditional medical screening methods. Within the realm of joint modeling, there exist two primary types of joint models for longitudinal and time-to-event data: shared parameter joint models [43,53,75,77] and joint latent class models [35,47,48,63]. In this work we focus our attention on the shared parameter joint models. One notable benefit of utilizing shared parameter joint models is their capacity to seamlessly integrate individual variability by employing random effects. Additionally, they provide the capability to generate individualized dynamic predictions regarding the risk of future events based on the evolving patterns of repeatedly measured endogenous biomarkers over time [52].

Personalized screening strategies hold great potential in addressing healthcare challenges in South Africa, particularly for individuals with a history of drug-resistant tuberculosis (TB) or prior non-adherence to TB or antiretroviral treatment (ART) who are returning to the healthcare system. Personalized screening, facilitated by advanced statistical models like joint models, makes it particularly powerful in personalized screening, especially in high-risk populations like those with a history of drug-resistant tuberculosis (TB) or prior treatment non-adherence.

While joint models have been used in personalized screening for determining personalized screening intervals for patients undergoing aortic allograft root implantation [58], utilizing predictions from the joint model to inform optimal intervention timing for abdominal aortic aneurysm [67] and for personalized decision-making regarding biopsies in cases of prostate cancer [71–73], their application in TB/HIV screening, particularly in sub-Saharan Africa, remains underexplored. The unique epidemiological and clinical characteristics of HIV and TB in this region necessitate tailored screening methodologies that go beyond cancer-focused approaches. Simply adopting methodologies developed for cancer screening may not adequately address these unique dynamics and challenges.

To bridge this gap, we make three key contributions beyond the existing literature. First, we extend multivariate joint models to incorporate censored longitudinal outcomes, addressing gaps in the literature where left-censored longitudinal outcomes have been less explored [31,70]. We account for censoring using the censored mixed models proposed by Thiébaut and Jacqmin-Gadda [69], which handles both the continuous nature of the longitudinal outcomes and censoring due to detection limits within the same model, avoiding biases and loss of information from common modeling strategies that dichotomize the censored longitudinal outcomes [32,40].

Second, we employ super learning techniques for optimal model selection, addressing the limitations of traditional model selection methods (e.g. DIC, information criteria) that focus on model fit rather than predictive accuracy for event-free individuals [57]. Our work extends the foundational work by Rizopoulos and Taylor [57] by applying super learning techniques to select the optimal multivariate joint model while accounting for censored longitudinal outcomes.

Third, we introduce a novel risk-based framework for deriving personalized screening intervals using multivariate joint models. While previous research has focused on multivariate joint models with binary and continuous longitudinal outcomes [72,73], our approach accommodates multivariate joint models with censored longitudinal outcomes, offering a more flexible and accurate method for complex data scenarios.

The rest of the paper is organized as follows. In Section 2 we provide an overview of the data used in this paper. In Section 3, we provide a summary of the statistical analysis test conducted. Section 4 outlines the multivariate joint modeling framework. Section 5 discusses the super learning techniques employed in this paper. Section 6 describes the personalized scheduling methodology. Section 7 includes the application of the methods used as well as model fit assessments. Finally, Sections 8 and 9 concludes with a discussion of our findings and Section 10 outlines areas of future work.

## 2. Source of data and description

We used data from the Centre for the AIDS Programme of Research in South Africa (CAPRISA) AIDS Treatment programme, the Starting Antiretroviral Therapy at Three Points in Tuberculosis (SAPiT) study, an open-label, three arm randomized, controlled trial between 28 June 2005 and 04 July 2010. This trial sought to determine the best time to start antiretroviral therapy (ART) in patients receiving TB treatment who are also co-infected with HIV undergoing TB treatment in KwaZulu-Natal [1,2], a region characterized by elevated TB [20] and HIV prevalence in South Africa [29]. The timing of ART introduction during TB treatment in South Africa was not formally guided prior to this investigation and as such this study contributed to the subsequent clinical guidelines. More details about the study and the results for primary and secondary outcomes have been published in detail elsewhere [1,2,41,79].

## 3. Statistical analysis

Descriptive data was presented as means with standard deviations (SD) or medians with interquartile range (IQR) and percentages. All multivariable models were adjusted for the study arm (combined early and late integrated therapy arms versus sequential therapy arm) which aligns with the primary study findings, which showed no significant difference in rates of AIDS or death between patients receiving early and late integrated ART [1]. Combining the arms was also motivated by a subsequent paper by the same authors which showed that combining these two arms provides a clearer and more concise representation of the data without losing any critical insights [2], gender and age. The CD4 count was normalized using a square root transformation, while the viral load was log-transformed. Analyses were conducted using R version 4.1.2. The most recent JMbayes2 package [56] was used to fit the joint models. We used a Bayesian estimation procedure and a Markov chain Monte Carlo (MCMC) algorithm to fit the joint models because of its flexibility in dealing with complex models.

## 4. Multivariate joint models of longitudinal and time to event data

Consider a random sample $D_{n}=\{T_{i},T_{i}^{U},\delta_{i},y_{i};i=1,\ldots ,n\}$ drawn from the target population. Where $T_{i}^{*}$ signifies the true event time for the *i*-th subject and $T_{i}$ and $T_{i}^{U}$ denotes the observed event times, such that $\delta_{i}\in \{0,1,2,3\}$ denotes the event indicator with 0 corresponding to right-censoring $\left(T_{i}^{*}>T_{i}\right)$, 1 to a true event $\left(T_{i}^{*}=T_{i}\right)$, 2 to left-censoring $\left(T_{i}^{*}<T_{i}\right)$, and 3 to interval-censoring $\left(T_{i}<T_{i}^{*}<T_{i}^{U}\right)$. Additionally, let $y_{\mathrm{ki}}$ denote the $n_{\mathrm{ki}}\times 1$ longitudinal response vector for the *k*-th outcome ( k=1,…,K) and the *i*-th subject, with $y_{\mathrm{kij}}$ denoting the value of the *k*-th longitudinal outcome for the *i*-th subject, taken at time point $t_{\mathrm{kij}},j=\{1,\ldots ,n_{\mathrm{ki}}\}$.

### 4.1. The longitudinal sub-model

To accommodate various types of longitudinal responses, a generalized linear mixed-effects model (GLMM) is postulated. Specifically, we assume that the conditional distribution of the *k*-th outcome $y_{\mathrm{ki}}$, given a vector of random effects ${\text{b}}_{\mathrm{ki}}$, belongs to the exponential family. The linear predictor is given by:

(1) $$g_{k}\left[E\left\{y_{\mathrm{ki}}(t)\;|\;{\text{b}}_{\mathrm{ki}}\right\}\right]=m_{\mathrm{ki}}(t)={\text{x}}_{\mathrm{ki}}^{T}(t){\text{β}}_{k}+{\text{z}}_{\mathrm{ki}}^{T}(t)b_{\mathrm{ki}}.$$

Where, $g_{k}(\cdot )$ denotes a known one-to-one monotonic link function, and $y_{\mathrm{ki}}(t)$ denotes the value of the *k*-th longitudinal outcome for the *i*-th subject at time point *t*. The vectors ${\text{x}}_{\mathrm{ki}}(t)$ and ${\text{z}}_{\mathrm{ki}}(t)$ correspond to the time-dependent design vectors for the fixed effects ${\text{β}}_{k}$ and the random effects $b_{\mathrm{ki}}$, respectively. The random effects are assumed to follow a multivariate normal distribution with mean zero and a variance–covariance matrix D.

### 4.2. The survival sub-model

In the survival process, it is assumed that the risk for an event depends on a function of the subject-specific linear predictor $m_{i}(t)$ and/or the random effects as;

$$\begin{array}{rl} h_{i}(t\;|\;H_{i}(t),w_{i}(t)) & \;=\lim_{\mathrm{\Delta t}\to 0}\mathrm{Pr}\left\{t\leq T_{i}^{*}<t+\mathrm{\Delta t}\;|\;T_{i}^{*}\geq t,H_{i}(t),w_{i}(t)\right\}/\mathrm{\Delta t},\\ & \;=h_{0}(t)\exp\left[\gamma^{T}w_{i}(t)+\sum_{k=1}^{K}\sum_{l=1}^{L_{k}}f_{\mathrm{kl}}\left\{H_{\mathrm{ki}}(t),w_{i}(t),b_{\mathrm{ki}},\alpha_{\mathrm{kl}}\right\}\right],\;t>0 \end{array}$$

where $H_{i}(t)=\{m_{i}(s),0\leq s<t\}$ represents the historical information of the underlying longitudinal process up to time *t* and $h_{0}(\cdot )$ represents the baseline hazard function with $w_{i}(t)$ representing a vector of baseline exogenous covariates, featuring corresponding regression coefficients denoted by γ. The function $f_{\mathrm{kl}}(\cdot )$, which is parameterized by vector $\alpha_{\mathrm{kl}}$, defines the characteristics of the longitudinal outcome process that are incorporated into the linear predictor of the relative risk model. Some examples, motivated by the literature [53–55,68] where subscripts *kl* have been dropped for simplicity, but are assumed in the following expressions

$$f\left\{H_{i}(t),w_{i}(t),b_{i},\alpha \right\}=\left\{ \begin{array}{l} \alpha m_{i}(t),\\ \alpha m_{i}^{\prime }(t),\;m_{i}^{\prime }(t)=\frac{dm_{i}(t)}{dt},\\ \alpha \int_{0}^{t}m_{i}(s)\;ds,\\ \alpha^{T}{\text{b}}_{i}. \end{array}\right.$$

These formulations suggests that the hazard of an event at time *t* may be linked to the current underlying value of the biomarker at the same time point; the velocity or slope of the longitudinal profile at *t*; the area accumulated under the longitudinal process up to *t*, or the random effects alone. Lastly, to flexibly model the baseline hazard function $h_{0}(\cdot )$ a B-splines approach is employed, specifically

$$\log h_{0}(t)=\gamma h_{0,0}+\sum_{q=1}^{Q}\gamma h_{0,q}B_{q}(t,\text{v}).$$

Where, $B_{q}(t,\text{v})$ represents the *q*-th basis function of a B-spline with knots $v=v_{1},\ldots ,v_{Q}$, and $\gamma h_{0}$ denotes the vector of spline coefficients with $\gamma h_{0,0}$ representing the intercept. To avoid the need to choose the number and positions of the knots, we incorporate a relatively high number of knots (e.g. 15–20) and apply a penalty for smoothness to the B-spline regression coefficients $\gamma h_{0}$ using the differences penalty [17].

## 5. Leveraging the super learning ensemble to choose the suitable model at time *t*

Our goal is to determine the optimal time to schedule the future visit to obtain biomarker measurements in the same population, such that [k,1] is the interval in which the subject's probability of surviving at a given time point *t* declines. As we have just seen, developing a joint model involves selecting suitable models for the longitudinal outcome (including baseline covariates and the functional form of the time effect), the time-to-event outcome (including baseline covariates), and determining how to link the two processes using the functional forms in f(⋅). Consequently, a pivotal question emerges: which model should be utilized to determine the timing of the next measurement for a new subject? Answering this question can be challenging as it is a complex task to select a well-specified model, particularly when dealing with multivariate longitudinal outcomes. Additionally, the dynamic nature of predictions introduces complexity, as different models may exhibit varying levels of predictive accuracy at various follow-up visit times. Traditional model selection approaches such as the DIC (Deviance information criterion) and information criteria and (pseudo-) Bayes factors, may not be appropriate in identifying the best model that has best predictive capabilities for future events as they inherently offer overall assessments of a model's predictive ability, whereas we are interested in the model that best predicts future events given the fact that subject *j* was event-free up to time point *t*. To this end, to make a proper selection of the optimal model, we adopt multiple joint models with different expressions for the time effect on the longitudinal outcome and various functional forms to establish the connection between this outcome and the event process and integrate their dynamic predictions to optimize predictive accuracy. This is achieved through the application of super learning (SL) [9,42,46,76]. SL, an ensemble method, enable us to blend diverse prediction algorithms into a cohesive framework. We aim to extend the foundational work by Rizopoulos and Taylor [57], who applied super learning techniques to optimize dynamic predictions from univariate joint models. We extend their work by accommodating multivariate censored longitudinal outcomes. To achieve this, we utilize *V*-fold cross-validation. This method allows for the creation of an optimal combination of predictions from various candidate algorithms. The optimization process is driven by a user-defined objective function, which could involve minimizing mean squared error or maximizing the area under the receiver operating characteristic curve (AUROC). This strategy ensures the creation of a cohesive and effective predictive modeling framework.

### 5.1. Estimation of model weights

The fundamental concept behind super learning involves determining optimal model weights through cross-validated predictions. Let $L=\{M_{1},\ldots ,M_{L}\}$ denote a library of *L* models, encompassing a diverse set of potential models with no specific restrictions. This library can include models with variations in aspects such as the specification of time trends in the longitudinal sub-models and the functional form of the survival sub-model. To achieve this, the $D_{n}$ which is an original dataset is divided into *V* folds, with the choice of selecting *V* depending on the number and size of events in this dataset $D_{n}$ [46], ensuring that each fold contains an adequate number of events for robust predictive performance evaluation.

Using cross-validation method, the *L* models are fitted on the combined *v*−1 folds using hold-out validation which generates predictions for the *v*-th fold that has been left out. Optimal weights are determined for different follow-up times, considering the dynamic nature of predictions. In particular, for a sequence of time points $t_{1},\ldots ,t_{Q}$, where the number and placement of these time points should account for available event information in $D_{n}$, we define $R(t_{q},v)$ to represent subjects at risk at time $t_{q}$ within the *v*-th fold. To obtain predictions that are cross-validated for all subjects in $R(t_{q},v)$ we consider the following computation equation

$${\hat{\pi }}_{i}^{\left(v\right)}(t_{q}+\mathrm{\Delta t}\;|\;t_{q},M_{l})=\mathrm{Pr}\left\{T_{i}^{*}<t_{q}+\mathrm{\Delta t}\;|\;T_{i}^{*}>t_{q},H_{i}(t),M_{l},D_{n}^{\left(-v\right)}\right\}.$$

The predictions are generated utilizing model $M_{l}$ from the library L, which was trained on the dataset $D_{n}^{\left(-v\right)}$ excluding the patients in the *v*-th fold. This computation employs a Monte Carlo approach [52], and ${\hat{\tilde{\pi }}}_{i}^{v}(t_{q}+\mathrm{\Delta t}\;|\;t_{q})$ represents the convex combination of the *L* predictions as;

$${\hat{\tilde{\pi }}}_{i}^{v}(t_{q}+\mathrm{\Delta t}\;|\;t_{q})=\sum_{l=1}^{L}\varpi_{l}(t_{q}){\hat{\pi }}_{i}^{\left(v\right)}(t_{q}+\mathrm{\Delta t}\;|\;t_{q},M_{l}),\;\mathrm{for}\;\mathrm{all}\;v\in \{1,\ldots ,V\}.$$

where $\varpi_{l}>0$ for l=1,…,L and $\sum_{l}\varpi_{l}(t_{q})=1$. It's important to note that the weights ϖ(⋅) are time-dependent, meaning that at different follow-up times, different combinations of the *L* models may result in more accurate predictions.

### 5.2. Evaluating predictive accuracy

For any given time *t*, we choose the weights $\left\{\varpi_{l}(t);l=1,\ldots ,L\right\}$ that optimize the predictive performance of the combined cross-validated predictions using proper scoring rules. A scoring rule $S\{\pi_{i}(u\;|\;t),I(t<T_{i}^{*}<u)\}$ is considered proper if the true distribution achieves the optimal expected score. In our case, this involves comparing the true conditional risk probabilities $\pi_{i}^{\mathrm{true}}(u\;|\;t)$ to the estimated probabilities ${\hat{\pi }}_{i}(u\;|\;t)$. The scoring rule S(⋅,⋅) is structured in such a way that a reduced score signifies enhanced accuracy., i.e

$$E\left[S\left\{\pi_{i}^{\mathrm{true}}(u\;|\;t),I(t<T_{i}^{*}<u)\right\}\right]\leq E\left[S\left\{{\hat{\pi }}_{i}(u\;|\;t),I(t<T_{i}^{*}<u)\right\}\right],\;u>t$$

The calculation involves considering the expectation with respect to the conditional density of the survival outcome under the true model $\left\{T_{i}^{*}\;|\;T_{i}^{*}>t,Y_{i}(t)\right\}$ where $Y_{i}(t)=\{y_{i}(t_{\mathrm{il}})\;|\;0\leq t_{\mathrm{il}}\leq t,l=1,\ldots ,n_{i}\}$.

### 5.3. The brier score

The Brier Score (BS) serves as a proper scoring rule, evaluating the accuracy of probabilistic predictions by simultaneously quantifying discrimination and calibration performances [19]. A lower Brier score reflects better calibration, and this metric encompasses both aspects of model performance [22]. Good discrimination in predictive models is prioritized over calibration. However, poorly calibrated models can be adjusted to enhance predictive accuracy. Despite this, both components are considered in the Brier Score [14]. In the context of dynamic risk prediction, time-dependent versions of predictive accuracy measures are utilized. Specifically, at the follow-up time *t* within a clinically significant time interval Δt, the Brier score is defined as follows:

$$\mathrm{BS}(t,t+\mathrm{\Delta t})=E\left[{\left\{I(T_{i}^{*}\leq t+\mathrm{\Delta t})-{\hat{\tilde{\pi }}}_{i}^{v}(t+\mathrm{\Delta t}\;|\;t)\right\}}^{2}|T_{i}^{*}>t\right].$$

To estimate the Brier score BS(t,t+Δt), it is necessary to properly account for censored individuals in the interval (t,t+Δt] [57]. There are two methods for achieving this, one involves using inverse probability of censoring weights (IPCW) and the other uses model-based weights. In the IPCW approach, only subjects with an event before t+Δt and those for whom survival up to t+Δt is known are considered. Patients censored in (t,t+Δt] do not contribute in this method [8]. In other words, we exclusively incorporate individuals with known outcomes up to the specified time points in the analysis.

$${\hat{\mathrm{BS}}}_{\mathrm{IPCW}}(t,t+\mathrm{\Delta t})=\frac{1}{n_{t}}\sum_{i:T_{i}>t}{\hat{W}}_{i}(t,t+\mathrm{\Delta t}){\left\{I(T_{i}\leq t+\mathrm{\Delta t})-{\hat{\tilde{\pi }}}_{i}^{v}(t+\mathrm{\Delta t}\;|\;t)\right\}}^{2}$$

where, $n_{t}$ represents the number of individuals at risk at time *t* with

$$\begin{array}{rl} {\hat{W}}_{i}(t,t+\mathrm{\Delta t}) & \;=\frac{I(t<T_{i}\leq t+\mathrm{\Delta t})\delta_{i}}{\hat{G}(T_{i}\;|\;t)}+\frac{I(t>T_{i}+\mathrm{\Delta t})}{\hat{G}(t+\mathrm{\Delta t}\;|\;t)}\\ & \;\;+0\cdot I(t<T_{i}\leq t+\mathrm{\Delta t})(1-\delta_{i}), \end{array}$$

where $\hat{G}(\cdot )$ defines the Kaplan-Meier estimate of the censoring distribution $\mathrm{Pr}(C_{i}>t)$. The model-based approach as defined by Henderson *et al.* [24] incorporates all individuals who are at risk at time *t*, specifically

$$\begin{array}{rl} {\hat{\mathrm{BS}}}_{\mathrm{model}} & \;=\frac{1}{n_{t}}\sum_{i:T_{i}>t}\delta_{i}I(T_{i}\leq t+\mathrm{\Delta t}){\left\{1-{\hat{\tilde{\pi }}}_{i}^{v}(t+\mathrm{\Delta t}\;|\;t)\right\}}^{2}\\ & \;\;+I(t>T_{i}+\mathrm{\Delta t}){\left\{{\hat{\tilde{\pi }}}_{i}^{v}(t+\mathrm{\Delta t}\;|\;t)\right\}}^{2}\\ & \;\;+(1-\delta_{i})I(T_{i}\leq t+\mathrm{\Delta t})[{\hat{\tilde{\pi }}}_{i}^{v}(t+\mathrm{\Delta t}\;|\;T_{i}){\left\{1-{\hat{\tilde{\pi }}}_{i}^{v}(t+\mathrm{\Delta t}\;|\;t)\right\}}^{2}\\ & \;+\left\{1-{\hat{\tilde{\pi }}}_{i}^{v}(t+\mathrm{\Delta t}\;|\;T_{i})\right\}{\left\{{\hat{\tilde{\pi }}}_{i}^{v}(t+\mathrm{\Delta t}\;|\;t)\right\}}^{2}]. \end{array}$$

The two approaches outlined above (i.e, IPCW and model-based approaches) each come with their own strengths and weaknesses. The IPCW approach is advantageous in that it is model-free, meaning it does not assume the correctness of the joint model used for computing ${\hat{\pi }}^{\left(v\right)}(t_{q}+\mathrm{\Delta t}\;|\;t_{q},M_{l})$. However, it makes a robust assumption that censoring is independent of covariates and, critically, the history of the longitudinal biomarker $Y_{i}(t)$. This assumption is pivotal, as the Brier score is a proper scoring rule only when this assumption holds [51]. If an inaccurate assumption is made regarding censoring weights, there is a risk of achieving an incorrect Brier score, potentially lower than the true weights [33]. In clinical contexts, where physicians may exclude patients based on observed longitudinal measurements, specifying an appropriate model for the censoring process becomes challenging, diminishing the appeal of the IPCW approach.

On the other hand, the model-based weights approach doesn't necessitate modeling the censoring process, making it robust to complex dependencies on the longitudinal history. However, it requires a well-specified joint model [57]. Comparing multiple models becomes paradoxical, as all models must be correctly specified for fair comparisons. Yet, in the context considered here, where the goal is to combine predictions for improved accuracy rather than comparing models, the model-based approach's disadvantages are mitigated. The weights for censored subjects are derived from a convex combination of the weights from multiple models, and cross-validated predictions ensure down-weighting of predictions from over-fitted models.

### 5.4. The expected predictive cross-entropy

We also consider an alternative scoring rule proposed by Commenges *et al.* [12] within the interval (t,t+Δt], which is a modified version of the expected predictive cross-entropy (EPCE) given by

$$\mathrm{EPCE}(t,t+\mathrm{\Delta t})=E\left\{-\log\left[p\{T_{i}^{*}\;|\;t<T_{i}^{*}<t+\mathrm{\Delta t},Y_{i}(t),D_{n}\}\right]\right\},$$

with the expectation taken with respect to $\left\{T_{i}^{*}\;|\;T_{i}^{*}>t,Y_{i}(t)\right\}$ under the true model. To obtain an estimate for the EPCE that accommodates censoring, we use the available sample, such that

$$E\hat{\mathrm{PC}}E(t,t+\mathrm{\Delta t})=\frac{1}{n_{t}}\sum_{i:T_{i}>t}-\log\left[p\left\{{\tilde{T}}_{i},{\tilde{\delta }}_{i}\;|\;T_{i}>t,Y_{i}(t),D_{n}\right\}\right],$$

where, ${\tilde{T}}_{i}=\min(T_{i},t+\mathrm{\Delta t})$ and ${\tilde{\delta }}_{i}=\delta_{i}I(T_{i}\leq t+\mathrm{\Delta t})$. The EPCE makes similar assumptions about censoring as the model-based weights for the Brier Score at the same time points, assuming independence between the true event time $T_{i}^{*}$ and censoring time $C_{i}$ given the longitudinal biomarker $Y_{i}(t)$. Unlike the Brier Score, the EPCE does not require model weights to address censoring, avoiding a particular disadvantage. Nevertheless, the EPCE is criticized for its lack of interpretability compared to the Brier Score. While the Brier Score estimates a well-defined population parameter–the mean squared distance between observed and expected outcomes–the EPCE's value lacks a straightforward interpretation.

To derive super-learning model-specific weights using the EPCE, it is necessary to express it as a function of dynamic predictions from a joint model by redefining $\pi_{i}(u\;|\;t,M_{l})$ as the dynamic subject-specific survival probabilities, such that

$$\pi_{i}(u\;|\;t,M_{l})=\mathrm{Pr}\left\{T_{i}^{*}>u\;|\;T_{i}^{*}>t,Y_{i}(t),D_{n},M_{l}\right\},$$

where *u*>*t*. Next, we express the conditional predictive log-likelihood (with the assumption of conditioning on $M_{l}$, although it is omitted for clarity in the subsequent expressions):

$$\begin{array}{rl} \log\left[p\left\{{\tilde{T}}_{i},{\tilde{\delta }}_{i}\;|\;T_{i}>t,Y_{i}(t),D_{n}\right\}\right] & \;={\tilde{\delta }}_{i}\log\left[h_{i}\left\{{\tilde{T}}_{i}\;|\;Y_{i}(t),D_{n}\right\}\right]\\ & \;\;+\log\frac{\mathrm{Pr}\left\{T_{i}^{*}>{\tilde{T}}_{i}\;|\;Y_{i}(t),D_{n}\right\}}{\mathrm{Pr}\left\{T_{i}^{*}>t\;|\;Y_{i}(t),D_{n}\right\}}. \end{array}$$

In the above expression, the second term on the right hand side of the equation represents $\log\{\pi_{i}({\tilde{T}}_{i}\;|\;t)\}$ and the hazard function $h_{i}(\cdot )$ in the first term can be expressed as

$$h_{i}\left\{{\tilde{T}}_{i}\;|\;Y_{i}(t),D_{n}\right\}=-\frac{\frac{d}{\mathrm{dt}}\mathrm{Pr}\left\{T_{i}^{*}>t\;|\;Y_{i}(t),D_{n}\right\}{|}_{t={\tilde{T}}_{i}}}{\mathrm{Pr}\left\{T_{i}^{*}>{\tilde{T}}_{i}\;|\;Y_{i}(t),D_{n}\right\}},$$

Merging these two terms results in the following:

$$E\hat{\mathrm{PC}}E(t,t+\mathrm{\Delta t})=-\frac{1}{n_{t}}\sum_{i:T_{i}>t}{\tilde{\delta }}_{i}\left[\log\{1-\pi_{i}({\tilde{T}}_{i}+\epsilon \;|\;{\tilde{T}}_{i})-\log(\epsilon )\}\right]+\log\{\pi_{i}({\tilde{T}}_{i}\;|\;t)\}.$$

In practical applications, $E\hat{\mathrm{PC}}E(t,t+\mathrm{\Delta t})$ can be calculated using a small value for *ϵ* for instance ϵ=0.001. Numerical experiments conducted indicate that the EPCE values are minimally influenced by the choice of *ϵ*. In scenarios demanding a more accurate approximation, the central difference approximation may be employed. This technique incurs a truncation error approximately proportional to $\epsilon^{2}$ but mandates an additional evaluation of the function.

In our scenario, both BS(t,t+Δt) and EPCE(t,t+Δt) are computed based on the convex combination of the cross-validated predictions ${\hat{\tilde{\pi }}}_{i}^{v}(t+\mathrm{\Delta t}\;|\;t)$. Specifically, through the super-learning process, we determine the weights ${\hat{\varpi }}_{l}(t)$ that minimize a proper scoring rule (either BS(t,t+Δt) or EPCE(t,t+Δt)) for the cross-validated predictions. Hence the weights are given by

$${\hat{\varpi }}_{l}(t)={\mathrm{argmin}}_{\varpi }\left[S\left\{\sum_{l=1}^{L}\varpi {\hat{\pi }}_{i}^{\left(v\right)}(t+\mathrm{\Delta t}\;|\;t,M_{l}),T_{i},\delta_{i}\right\}\right],\;v=1,\ldots ,V$$

while satisfying the constraints $\varpi_{l}(t)>0$ for l=1,…,L and $\sum_{l}\varpi_{l}(t)=1$.

## 6. Planning for the next measurement of CD4 count and viral load

After successfully selecting an optimal model, the next objective is to determine the ideal time to schedule the next biomarker measurement for a new subject. Despite the crucial role of personalized decisions in HIV monitoring, there is a lack of exploration in this area. To address this gap, our aim is to introduce innovative methodology for obtaining personalized screening intervals specifically tailored to HIV biomarkers.

### 6.1. The cumulative risk of death

Let us consider a scenario where a decision needs to be made regarding the frequency of monitoring (screening intervals) for a new patient with HIV, denoted as patient *j*. In particular, we let $t\leq T_{j}^{*}$ be the time of his latest visit where the patient's information includes CD4 count and viral load (VL) measurements at various follow-up time points up to the current visit time *v*, denoted by $Y_{\mathrm{CD}4}(v)$ and $Y_{\mathrm{VL}}(v)$, respectively. Let *t* be the last known time when the patient had stable CD4 count and viral load measurements. Stability here refers to a period where the patient's measurements were within a certain range that indicates good health or controlled disease. While *v* represents the current visit time, which is the most recent time point at which the patient's CD4 count and viral load were measured, regardless of their stability. Our goal entails estimating the underlying trend of CD4 count and viral load and use this combined information to determine the optimal time for the next screening visit. We will employ the posterior predictive distribution, merging information from the observed data of the of the *j*-th subject $\left\{T_{j}^{*}>t,Y_{\mathrm{CD}4}(v),Y_{\mathrm{VL}}(v)\right\}$ with the original dataset used for fitting the joint model $D_{n}$. This approach maintains a cross-validatory nature since j∉{i=1,…,n}. The posterior predictive distribution is expressed as;

$$g(T_{j}^{*})=p\left\{T_{j}^{*}\;|\;T_{j}^{*}>t,Y_{\mathrm{CD}4}(v),Y_{\mathrm{VL}}(v),D_{n}\right\}.$$

The distribution of $g(T_{j}^{*})$ is specifically tailored for each individual patient and adapts as additional data, such as CD4 count and viral load measurements, is collected during future follow-up visits.

We aim to calculate the personalized risk of death for a new patient *j* at time $T_{j}^{*}$. To achieve this, we propose a straightforward approach, which is to compare the risk to a predefined threshold, denoted as 0≤k≤1. A reasonable choice is to set *k* equal to the constant c(t) to decide whether intervention by the physician is necessary. The determination of c(t) can be based on medical considerations, such as ensuring that cumulative risk probabilities do not exceed a specific threshold. For example, if the risk exceeds a 5% threshold, it may be advisable to conduct additional tests. The threshold can be adjusted based on the preferences of both the patient and the healthcare provider. Using the fitted joint model, one can obtain the cumulative risk of progression for a new patient *j* at a future time *u* by extracting information from the posterior predictive distribution $g(T_{j}^{*})$. This approach, applied within the framework of dynamic individualized predictions, is documented in the literature [52,53,68,80], as;

$$\begin{array}{rl} R_{j}(u\;|\;t,v) & \;=\mathrm{Pr}\left\{T_{j}^{*}\leq u\;|\;T_{j}^{*}>t,Y_{\mathrm{CD}4}(v),Y_{\mathrm{VL}}(v),D_{n}\right\}\\ & \;=\int \int \mathrm{Pr}(T_{j}^{*}\leq u\;|\;T_{j}^{*}>t,b_{j},\theta )\\ & \;\;\times p\left\{b_{j}\;|\;T_{j}^{*}>t,Y_{\mathrm{CD}4}(v),Y_{\mathrm{VL}}(v),\theta \right\}\\ & \;\;\times p(\theta \;|\;D_{n})\;db_{j}\;d\theta ,\;u\geq t \end{array}$$

where $T_{j}^{*}$ represents the time of death for patient *j* with CD4 counts dropping below a certain threshold (i.e. CD4 count below 200 cells/mm^3^), indicating a deterioration in immune function and progression to more advanced stages of HIV disease. $T_{j}^{*}>t$ indicates that death has not occurred up to time *t*. The decision-making process is iterative and continues over the patient's follow-up period. The cumulative-risk function $R_{j}(\cdot )$ is dynamic, meaning that at each subsequent visit, the model is updated as new CD4 count and viral load data becomes available. The personalized risk $R_{j}(\cdot )$ represents the probability of the patient's health deteriorating (e.g. a significant drop in CD4 count or a spike in viral load) between time *t* and future time *u*. This risk assessment incorporates the patient's historical data and updates as new data becomes available. The decision to conduct additional screening such as CD4 count and viral load tests, during the scheduled follow-up visit *v* is contingent upon the personalized risk, considering that the patient remained alive until *u*.

### 6.2. Personalized test criterion

In order to enhance the chances of patients' long-term survival while simultaneously reducing the expenses and potential hazards linked to screening, medical professionals are keenly interested in monitoring events that occur within a clinically significant timeframe ( t,t+Δt]. In situations where the likelihood of a patient surviving beyond the time t+Δt falls below the predetermined threshold *k*, it becomes imperative for the physician to promptly intervene, aiming to enhance the patient's overall outlook. Conversely, when the probability of survival beyond t+Δt exceeds *k*, the physician may choose not to initiate treatment. Instead, their objective may be to enhance their comprehension of disease progression by integrating additional biomarker measurements. The decision to seek for an additional measurement demands a comprehensive assessment of the benefits and drawbacks for both the patient and the healthcare system. Consequently, our focus is on meticulously planning the timing of the next measurement to optimize the insights we can gather regarding disease progression while simultaneously reducing costs and minimizing the burden on the patient.

Our aim is to effectively adapt and extend the methodology introduced by Tomer *et al.* [72] to account for multivariate longitudinal outcomes with limit of detection issues. Our overarching goal is to develop a personalized, risk-based schedule for follow-up visits, specifically focusing on CD4 count and viral load measurements for individuals with HIV. This scheduling is guided by the cumulative-risk function $R_{j}(\cdot )$. We meticulously plan these follow-up visits based on the unique healthcare needs of each patient. Representing the sequence of visits as $U=\{u_{1},\ldots ,u_{L}\}$, where $u_{1}=v$ denotes the current visit time, we ascertain the furthest future visit time $u_{L}$ by leveraging information from the training dataset An. The planning extends up to $u_{L}$, ensuring a sufficient number of events in $A_{n}$ are available for reliable risk predictions. This optimization allows us to determine the most appropriate timing for the next CD4 count and viral load measurements. Our approach to planning is patient-cantered, acknowledging the unique healthcare requirements of each individual. In line with this, we suggest requesting a measurement at a future visit $u_{l}\in U$ if, at that specific time $u_{l}$, the cumulative risk of progression $R_{j}(\cdot )$ exceeds a predefined risk threshold *k*. This recommendation aligns with our objective of tailoring healthcare interventions based on the personalized risk profiles of HIV-positive patients. This iterative process ensures that the patient's screening intervals are personalized and optimized based on their unique health profile. Transparent communication between patients and healthcare providers is key to setting an appropriate risk threshold. Patients may have different preferences when balancing the risk of not detecting early signs of health deterioration against the inconvenience of frequent testing. Involving patients in the decision-making process empowers them to make informed choices about their healthcare. Given this consideration, the criterion/choice to perform the next measurement at time $u_{l}$ is expressed as

(2) $$Q_{j}^{k}(u_{l}\;|\;t_{l},v)=I\left\{R_{j}(u_{l}\;|\;t_{l},v)\geq k\right\},\;0\leq k\leq 1,$$

where the indicator function is denoted as I(⋅), and $R_{j}(u_{l}\;|\;t_{l},v)$ represents the cumulative risk of death at the current decision time $u_{l}$, where $t_{l}<u_{l}$ indicates the time where the last measurement was taken before $u_{l}$. Consequently, the determination of when a future measurement should be scheduled hinges on two things: i) the predefined threshold *k* and ii) the cumulative risk of death of the specific patient. Furthermore, if a measurement is scheduled at time $u_{l}$, denoted by $Q_{j}^{k}(u_{l}\;|\;t_{l},v)=1$, the cumulative-risk profile is adjusted before making the subsequent measurement decision at time $u_{l+1}$. Specifically, the cumulative risk at time $u_{l+1}$ is modified by setting the corresponding time of the last measurement as $t_{l+1}=u_{l}$. This adjustment takes into consideration the potential for progression occurring after time $u_{l}<T_{j}^{*}$. Consequently, the definition of the time of the last measurement $t_{l}$ is given by,

$$t_{l}=\left\{ \begin{array}{ll} t, & \mathrm{if}\;l=1,\\ u_{l-1}, & \mathrm{if}\;l\geq 2\;\mathrm{and}\;Q_{j}^{k}(u_{l-1}\;|\;t_{l-1},v)=1,\\ t_{l-1}, & \mathrm{if}\;l\geq 2\;\mathrm{and}\;Q_{j}^{k}(u_{l-1}\;|\;t_{l-1},v)=0. \end{array}\right.$$

### 6.3. Personalized schedules

If a new patient *j* sustains a stable health condition from their last measurement taken at time *t* up to the maximum future visit time $u_{l}$, and taking into account the subset of future visit times within the set *U*, we utilize the testing decision specified in Equation (2) to derive personalized screening intervals for upcoming longitudinal measurements, as follows:

(3) $$\{s_{1},\ldots ,s_{N_{j}}\}=\{u_{l}\in U:Q_{j}^{k}(u_{l}\;|\;t_{l})=1\},\;N_{j}\leq L.$$

The personalized screening intervals in Equation (3) get updated dynamically as additional data is collected over follow-up. In the event where patient *j* maintains a stable health status between their last recorded measurement at time *t* and the given maximum future visit time $u_{L}$, as initially assumed, the entire set of planned measurements, denoted by $\left\{s_{1},\ldots ,s_{N_{j}}\right\}$, will be carried out. This comprehensive testing approach ensures the completion of all scheduled biomarker measurements, facilitating precise health monitoring. However, should the patients' health deteriorate at any point $T_{j}^{*}<u_{L}$, the number of tests conducted will be adjusted. This adjustment is contingent upon the patient's health condition at the time of progression, leading to a personalized testing regimen designed to closely monitor their evolving health status. In this section, we introduce the discrete random variable $N_{j}$, quantifying the number of tests conducted taking into account the real progression time $T_{j}^{*}$, in the following manner:

$$N_{j}(S_{j}^{k})=\left\{ \begin{array}{ll} 1, & \mathrm{if}\;t<T_{j}^{*}\leq s_{1},\\ 2, & \mathrm{if}\;s_{1}<T_{j}^{*}\leq s_{2},\\ \vdots & \\ N_{j}, & \mathrm{if}\;s_{N_{j}-1}<T_{j}^{*}\leq s_{N_{j}}. \end{array}\right.$$

where $S_{j}^{k}$ denotes the scheduled future tests.

## 7. Results

### 7.1. Exploratory data analysis

**Figure 2.**
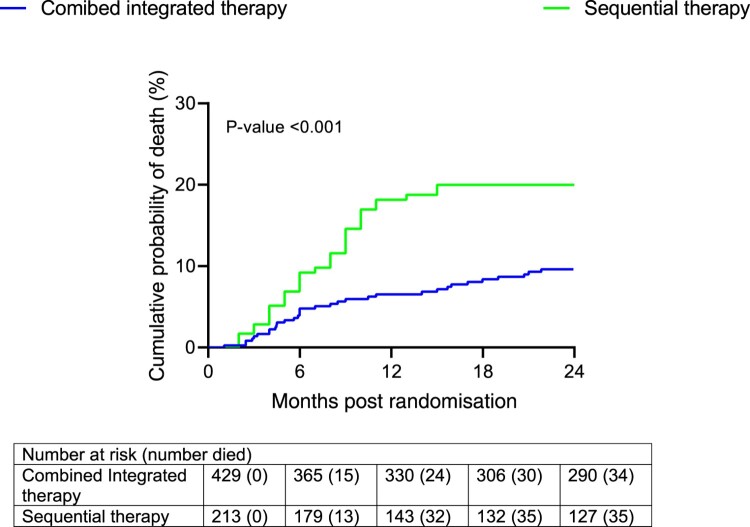
Cumulative risk.

**Figure 3.**
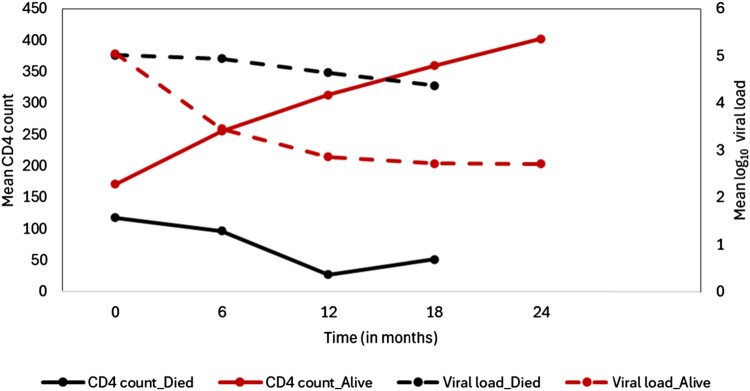
The evolution of the mean CD4 count (cells/mm^3^) and mean log_10_ viral load (copies/ml) over time.

The sequential treatment exhibited an elevated risk of mortality compared to integrated therapy, as illustrated in Figure 2. Throughout the follow-up duration, there were 69 recorded patient deaths, constituting 10.7% of the total. Among these, 34 were in the integrated therapy treatment group, while 35 were in the sequential treatment group (Table 1). Notably, a significant proportion of these deaths occurred within the initial 12 months after randomisation, precisely at 9.47 months (Table 1). In addition, participants that died had markedly low CD4 counts (median CD4 count: 67; IQR: 26–134) and exceptionally high viral loads (median viral load: 221000; IQR: 38050–497000), signaling rapid progression of HIV disease. This is also supported by Figure 3 which shows that those who died had significantly lower mean CD4 counts and higher mean log viral load over time. The longitudinal sub-model for CD4 count for the *i*-th subject, i=1,…,n, is defined as follows:

(4) $$\begin{array}{rl} y_{i}(t) & \;=({\text{β}}_{0}+b_{i0})+({\text{β}}_{1}+b_{i1})B_{1}(t,\lambda_{1})+({\text{β}}_{2}+b_{i2})B_{2}(t,\lambda_{2})+{\text{β}}_{3}{\mathrm{Arm}}_{i}+{\text{β}}_{4}{\mathrm{Age}}_{i}\\ & \;\;+{\text{β}}_{5}{\mathrm{Gender}}_{i}+\epsilon_{i}(t), \end{array}$$

where { $B_{n}(t,\lambda_{k}):k=1,2$} denotes the B-spline basis matrix for a natural cubic spline of time with one internal knot placed at 9 months post randomisation. The error $\epsilon_{i}(t)$ follows a normal distribution $N(0,R_{i})$ and the random effects ${\text{b}}_{i}$ follow a normal distribution N(0,D), with ${\text{R}}_{i}=\sigma_{\epsilon }^{2}{\text{I}}_{\mathrm{ni}}$ and D represents an unstructured variance-covariance matrix.

**Table 1.** Baseline characteristics of the study population stratified by death status.

Variable	Alive (*N* = 573)	Died (*N* = 69)	*P*-value
Mean age (SD), years	34.2 (8.4)	34.9 (7.7)	0.470
Number of males, n (%)	275 (48.0)	44 (63.8)	0.013
Median CD4+ count (IQR)	152 (77–248)	67 (26–134)	<0.001
Median viral load (IQR)	400 (400–39000)	221000 (38050–497000)	<0.001
Months at risk of death, median (IQR)	22.39 (22.29–22.62)	9.47 (6.21–15.25)	<0.001

**Figure 4.**
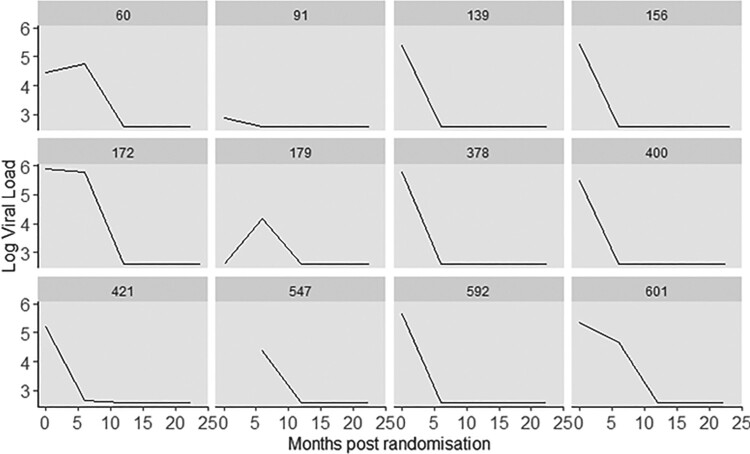
Patient-specific longitudinal trajectories of viral load (log_10_copies/ml) over time for 12 randomly selected patients.

Statistical modeling of viral load poses several challenges, including issues like heteroscedasticity, assay discordance, left censoring from lower limits of detection, correlation from repeated measurements, and the presence of missing data. Currently, all available assays come with a lower limit of quantification, typically within the range of 500 to 20 copies/ml, resulting in inherent left censoring of measurements. The extensive adoption of highly active antiretroviral treatments has resulted in a higher proportion of individuals exhibiting viral load levels that fall below a specified limit. Within the SAPIT dataset, the lower detection limit was set at 400 copies/ml. Figure 4 shows a nonlinear trend of viral load trajectories between and within participants. Therefore, careful consideration is crucial when selecting statistical methods for the analysis of longitudinally collected data. Neglecting to address censoring in the analysis may introduce significant biases in estimates related to outcome measurements [78], especially when the percentage of censoring is substantial. A commonly employed strategy to handle censored HIV-1 plasma RNA values is to substitute censored data with Limit of Detection (LOD) values or LOD/2 (particularly in cases of left censoring). Another approach involves analyzing outcomes on a binary scale by categorizing a measurement as either detectable or not detectable. Numerous methodologies have been developed to analyze such censored data [11,26,27,36,39]. To address censoring related to limits of detection issues, in this work, we utilized the censored linear mixed model [69]. In particular, let $Y_{\mathrm{ij}}$ denote the true viral load measurements for the *i*-th subject at time $t_{\mathrm{ij}}$ such that in the presence of censoring instead of $Y_{\mathrm{ij}}$ we observe the pair $\left(Y_{\mathrm{ij}}^{*},C_{\mathrm{ij}}\right)$, where $Y_{\mathrm{ij}}^{*}$ denotes the observed response value and $C_{\mathrm{ij}}\in \{0,1,2,3\}$ represents the censoring indicator such that $Y_{\mathrm{ij}}$ is observed if $C_{\mathrm{ij}}=0$ and is left-censored $\left(Y_{\mathrm{ij}}^{*}<d\right)$ if $C_{\mathrm{ij}}=1$, right-censored $\left(Y_{\mathrm{ij}}^{*}>d\right)$ if $C_{\mathrm{ij}}=2$ and interval-censored $\left(d_{L}<Y_{\mathrm{ij}}^{*}<d_{U}\right)$ if $C_{\mathrm{ij}}=3$, such that,

(5) $$Y_{\mathrm{ij}}=\left\{ \begin{array}{ll} Y_{\mathrm{ij}}^{*}, & \mathrm{if}\;C_{\mathrm{ij}}=0,\\ d, & \mathrm{if}\;C_{\mathrm{ij}}\in \{0,1,2,3\}, \end{array}\right.$$

where *d* denotes the lower limit of detection of the assay and $Y_{\mathrm{ij}}^{*}=x_{\mathrm{ij}}^{T}\text{β}+z_{\mathrm{ij}}^{T}b_{i}+\epsilon_{\mathrm{ij}}$, such that ${\text{x}}_{\mathrm{ij}}(t)$ and ${\text{z}}_{\mathrm{ij}}(t)$ correspond to the time-dependent design vectors for the fixed effects β and the random effects $b_{i}$, respectively. The model adjusts the likelihood function to accommodate censored data, where the exact viral load value is unknown but is below, above or within an interval of a certain detection limit. Specifically, let $f(y_{i}\;|\;b_{i})$ denote the density function of a mixed effects model given random effects $b_{i}$. Assuming that conditional on the random effects $b_{i}$, the observations $Y_{i1},Y_{i2},\ldots ,Y_{in_{i}}$ are independent, we can then define the likelihood for the observed data $\left(Y_{\mathrm{ij}}^{*},C_{\mathrm{ij}}\right)$ as

$$L(\theta )=\prod_{i=1}^{N}\int_{-\infty }^{\infty }\prod_{j=1}^{n_{i}}\left[{\left(f(y_{i}\;|\;b_{i})\right)}^{1-C_{\mathrm{ij}}}\times \left(F(d\;|\;b_{i}{)}^{C_{\mathrm{ij}}}\right)\right]f(b_{i})\;db_{i}.$$

The likelihood contribution for observed viral load values $\left(C_{\mathrm{ij}}=0\right)$ is given by

$$f(Y_{\mathrm{ij}}\;|\;b_{i})=\frac{1}{\sqrt{2\pi \sigma^{2}}}\exp\left(-\frac{(Y_{\mathrm{ij}}-(x_{\mathrm{ij}}^{T}\text{β}+z_{\mathrm{ij}}^{T}b_{i}){)}^{2}}{2\sigma^{2}}\right),$$

where $\sigma^{2}$ is the variance of the residual $\epsilon_{\mathrm{ij}}$.

*For left-censored observations $\left(Y_{\mathrm{ij}}^{*}<d\right)$*, the likelihood contribution is given by the cumulative distribution function (CDF) evaluated at the detection limit *d* as

$$P(Y_{\mathrm{ij}}^{*}<d)=\Phi \left(\frac{d-(x_{\mathrm{ij}}^{T}\text{β}+z_{\mathrm{ij}}^{T}b_{i})}{\sigma }\right),$$

where Φ(⋅) denotes the CDF of the standard normal distribution.

*For right-censored observations $\left(Y_{\mathrm{ij}}^{*}>d\right)$*, the likelihood contribution is given by 1-CDF as

$$P(Y_{\mathrm{ij}}^{*}<d)=1-\Phi \left(\frac{d-(x_{\mathrm{ij}}^{T}\text{β}+z_{\mathrm{ij}}^{T}b_{i})}{\sigma }\right).$$

*For interval-censored observations $\left(d_{L}<Y_{\mathrm{ij}}^{*}<d_{U}\right)$*, the likelihood contribution is the difference between the CDF at the upper and lower limits given by

$$P(d_{L}<Y_{\mathrm{ij}}^{*}<d_{U})=\Phi \left(\frac{d_{U}-(x_{\mathrm{ij}}^{T}\text{β}+z_{\mathrm{ij}}^{T}b_{i})}{\sigma }\right)-\Phi \left(\frac{d_{L}-(x_{\mathrm{ij}}^{T}\text{β}+z_{\mathrm{ij}}^{T}b_{i})}{\sigma }\right).$$

To account for non-linearity, we explored different mixed effects models with different specifications for time including quadratic, cubic and natural cubic splines. Using the BIC, we found the best model to be the one with natural cubic splines of time with one internal knot placed at 9 months post randomisation. The model also included the main effects of age, gender and treatment arm given by

(6) $$\begin{array}{rl} Y_{\mathrm{ij}}(t) & \;=({\text{β}}_{0}+b_{i0})+({\text{β}}_{1}+b_{i1})B_{1}(t,\lambda_{1})+({\text{β}}_{2}+b_{i2})B_{2}(t,\lambda_{2})+{\text{β}}_{3}{\mathrm{Arm}}_{i}+{\text{β}}_{4}{\mathrm{Age}}_{i}\\ & \;\;+{\text{β}}_{5}{\mathrm{Gender}}_{i}+\epsilon_{i}(t), \end{array}$$

where the model parameters follow a similar interpretation as in Model (4).

### 7.2. Leveraging the super learning ensemble to choose the suitable multivariate joint model at time *t*

To effectively select the optimal model to use at *t*, we examined five joint models and incorporated their dynamic predictions to enhance accuracy through super learning. These models were trained on parallel computing, facilitated by the parallel package in R. To objectively evaluate the predictive performance of each model with the available data $D_{n}$, we utilized *V*-fold cross-validation, holding out a fold during each iteration. Cross-validated predictions were then computed for the omitted fold, and an optimally weighted combination of predictions from various candidate algorithms was created. To evaluate the predictive performance of the five models, we considered three time intervals (t,t+Δt] that fall within the 24 month follow-up period for our data, namely (0.5,1], (0.5,1.25] and (0.5,1.5] where 0.5, 1.25 and 1.5 denotes 6 months, 15 months and 18 months follow-up time points respectively. At follow-up month 6, there were 543 patients still at risk and there were 30, 32 and 37 patients who died in the interval (0.5,1], (0.5,1.25] and (0.5,1.5] respectively. The weights were determined to optimize both the integrated Brier score and the expected predictive cross-entropy at these follow-up time intervals. The Jmbayes2 package [56] was used to fit the five multivariate joint models for the two outcomes (i.e. CD4 count and viral load) with different association structures taking the following functional forms

$$\begin{array}{rl} \mathrm{Model}1:\; & h_{i}(t)=h_{0}(t)\exp\left[\gamma^{T}w_{i}(t)+\sum_{k=1}^{K}\alpha_{k}\eta_{\mathrm{ki}}(t)\right],\\ \mathrm{Model}2:\; & h_{i}(t)=h_{0}(t)\exp\left[\gamma^{T}w_{i}(t)+\sum_{k=1}^{K}\alpha_{k}\eta_{\mathrm{ki}}^{\prime }(t)\right],\\ \mathrm{Model}3:\; & h_{i}(t)=h_{0}(t)\exp\left[\gamma^{T}w_{i}(t)+\sum_{k=1}^{K}\alpha_{k}\int_{0}^{t}\eta_{\mathrm{ki}}(s)\;ds\right],\\ \mathrm{Model}4:\; & h_{i}(t)=h_{0}(t)\exp\left[\gamma^{T}w_{i}(t)+\sum_{k=1}^{K}\alpha_{1k}\eta_{\mathrm{ki}}(t)+\sum_{k=1}^{K}\alpha_{2k}\eta_{\mathrm{ki}}^{\prime }(t)\right],\\ \mathrm{Model}5:\; & h_{i}(t)=h_{0}(t)\exp\left[\gamma^{T}w_{i}(t)+\sum_{k=1}^{K}\alpha_{1k}\eta_{\mathrm{ki}}(t)+\sum_{k=1}^{K}\alpha_{2k}\int_{0}^{t}\eta_{\mathrm{ki}}(s)\;ds\right]. \end{array}$$

Model 1 included the current value of the log viral load and current value of the square root CD4 count. While Model 2 included the current velocity/slope for each of the two longitudinal biomarkers. Model 3 included the area under each of the two longitudinal biomarkers. Model 4 included the current value plus the current slope for each longitudinal biomarker and lastly Model 5, included the current value plus the area under each of the two longitudinal biomarkers. Where the regression coefficients $\gamma^{T}=\{\gamma_{1},\gamma_{2},\gamma_{3}\}$ corresponds to a vector of baseline covariates $w_{i}=\{{\mathrm{Age}}_{i},{\mathrm{Women}}_{i},{\mathrm{Arm}}_{i}\}$ respectively.

Tables 2 and 3 presents results for super learning weights that combined the dynamic predictions from these models to optimize the two prediction metrics and calculated the two metrics using the weighted predictions. There integrated brier scores are very similar within the three intervals with minimal differences between them. The model with the area functional form has lower scores in the first and third intervals which is supported by higher model weights. In the interval (0.5,1] and (0.5,1.5] the weights are 0.7314 and 0.7844 respectively, which is higher than the other four models. We note that, in the (0.5,1.25] interval, the weights are dominated by the model with the current value plus slope functional form. The EPCE results in Table 3 are in line with Table 2, where the model with the area function contains the smallest EPCE score across the three time intervals, indicating improved predictive accuracy compared to the other four models. Considering that lower scores and higher weights signify better accuracy, we chose the multivariate joint model with the area functional form as the best model for our data. This decision is substantiated by its consistently lower scores for both the integrated Brier score and EPCE, along with its dominance in the IBS weights. Therefore, this chosen model will be employed for all calculations to derive personalized screening intervals in the subsequent sections.

**Table 2.** Estimated integrated brier score (IBS) for the five joint models along with their combination using super learning.

	(t,t+Δt]=(0.5,1]		(t,t+Δt]=(0.5,1.25]		(t,t+Δt]=(0.5,1.5]	
Models	IBS	Weights	IBS	Weights	IBS	Weights
SL	0.0256		0.0348		0.0445	
Value	0.0256	0.0819	0.0349	0.0396	0.0446	0.0101
Slope	0.0269	0.0783	0.0377	0.0011	0.0491	0
Area	0.0256	0.7314	0.0349	0.2679	0.0446	0.7844
Value+Slope	0.0259	0.1076	0.0348	0.6896	0.0449	0.2048
Value+Area	0.0266	0.1465	0.0361	0.0018	0.0460	0.1099

**Table 3.** Expected predictive cross-entropy (EPCE) for the five joint models along with their combination using super learning.

	(t,t+Δt]=(0.5,1]		(t,t+Δt]=(0.5,1.25]		(t,t+Δt]=(0.5,1.5]	
Models	EPCE	Weights	EPCE	Weights	EPCE	Weights
SL	0.1561		0.1728		0.2029	
Value	0.1590	0.1905	0.1761	0.1739	0.2068	0.1483
Slope	0.1824	0.2421	0.2035	0.2140	0.2387	0.1672
Area	0.1590	0.2536	0.1761	0.1743	0.2068	0.2564
Value+Slope	0.1631	0.2467	0.1785	0.3775	0.2084	0.4173
Value+Area	0.1648	0.067	0.1814	0.0603	0.2133	0.0177

Table 4 provides estimates of parameters and their corresponding 95% credibility intervals for the event process. After adjusting for baseline predictors, the association parameters estimated in Model 3 (area functional form) reveal significant associations between the risk of death and both the area under the longitudinal profile of CD4 count and viral load. Specifically, an increase in the area under the square root CD4 count by one unit corresponds to a 13% reduction in the hazard of death (HR=0.87; 95% CI: 0.80–0.94). While a unit increase in the area under the viral load profile corresponds to a 2.83-fold increase in the risk of death (HR=2.83; 95% CI: 1.79–5.25) (Table 4).

**Table 4.** Hazard ratios and their corresponding 95% credibility intervals (CI) for the parameters within the survival sub-models of five multivariate joint models.

	Current value	Slope	Area	Value+Slope	Value+Area
Variable	HR (95% CI)	HR (95% CI)	HR (95% CI)	HR (95% CI)	HR (95% CI)
Age	0.99 (0.95–1.05)	1.01 (0.95–1.07)	0.99 (0.94–1.06)	0.99 (0.94–1.05)	0.99 (0.94–1.06)
Integrated therapy	1.41 (0.77–2.77)	0.35 (0.14–0.73)	1.78 (0.93–3.80)	1.24 (0.65–2.69)	1.71 (0.81–4.39)
Women	0.54 (0.29–0.99)	0.34 (0.15–0.73)	0.57 (0.30–1.03)	0.52 (0.27–1.06)	0.55 (0.29–1.01)
$\alpha_{1}$	0.86 (0.78–0.94)	2.46 (1.51–4.02)	0.87 (0.80–0.94)	0.85 (0.76–0.94)	0.89 (0.57–1.38)
$\alpha_{2}$	1.89 (1.34–2.86)	2.61 (1.63–4.31)	2.83 (1.79–5.25)	1.16 (0.48–2.40)	0.96 (0.60–1.38)
$\alpha_{3}$				1.82 (1.33–2.71)	1.50 (0.71–2.71)
$\alpha_{4}$				1.07 (0.46–1.69)	1.63 (0.57–5.31)

### 7.3. Assessing model fit and convergence

In this work, the joint models were fitted under a Bayesian approach using Markov chain Monte Carlo algorithms because of its flexibility in handling complex models [61,65]. Assessing convergence in MCMC simulations is crucial to ensure the reliability of parameter estimates. To determine that the convergence has occurred for a model, one should check how the Markov chain is moving around the state space, that is, how well it is mixing. Various tools in the literature [10,13,21,62], provide methods to evaluate MCMC convergence and offer valuable feedback on the stability of the estimates.

In this study, we employed trace plots and kernel density plots to assess both convergence and model fit. Trace plots, also known as time-series plots, depict the sampled values of model parameters over successive MCMC iterations. They allow us to monitor how efficiently the Markov chain explores the parameter space and converges to a stationary distribution. A desirable outcome in trace plots is a ‘lazy caterpillar’ pattern, where parameter estimates remain within a reasonably narrow range across iterations without abrupt deviations [21].

Kernel density plots are essential tools in Bayesian analysis for visually assessing the distribution of parameter estimates derived from MCMC sampling [21]. These plots depict the smoothed probability density function (PDF) of estimated solutions across the simulated samples, offering insights into the central tendency, variability, and shape of the posterior distribution. A key expectation of kernel density plots is unimodality, meaning they typically display a single peak that represents the most probable values of the parameters [21]. This characteristic indicates that the Bayesian model has effectively captured the central tendency of the data, providing a clear indication of where the parameter estimates cluster. Additionally, kernel density plots are expected to have small tails, indicating that the probability density decreases rapidly in the extreme values of the parameter estimates [21]. Small tails suggest that the model's estimates are precise and reliable, with minimal variability in extreme scenarios, which enhances confidence in the robustness of the Bayesian inference. Moreover, the smoothness of kernel density plots further contributes to their utility by reducing noise and presenting a coherent representation of the parameter distribution [21].

**Figure 5.**
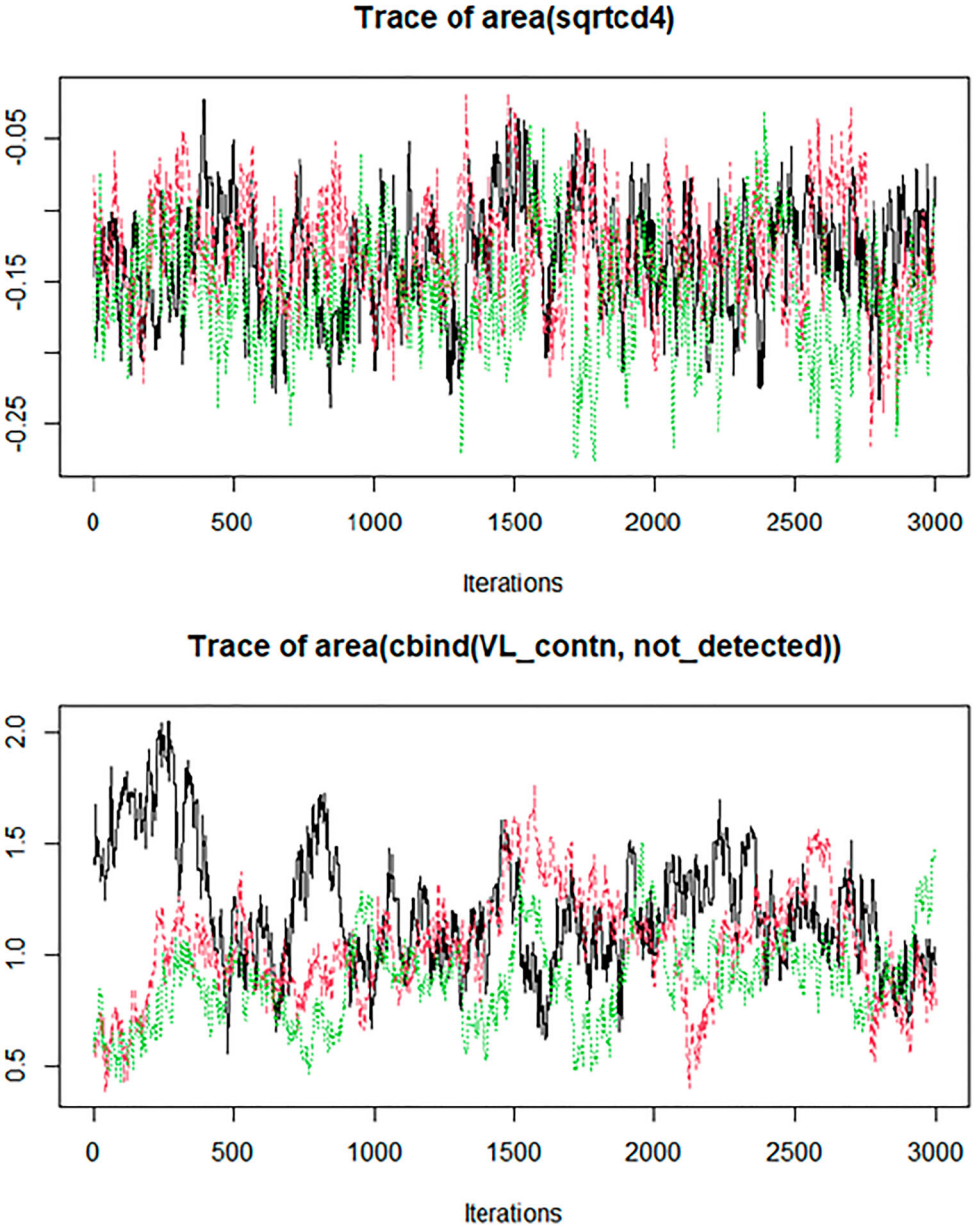
Trace plots for the area under the longitudinal profiles of log viral load and square root CD4 count with three chains of 3000 iterations. (a) Trace plot for CD4 count (b) Trace plot for viral load.

**Figure 6.**
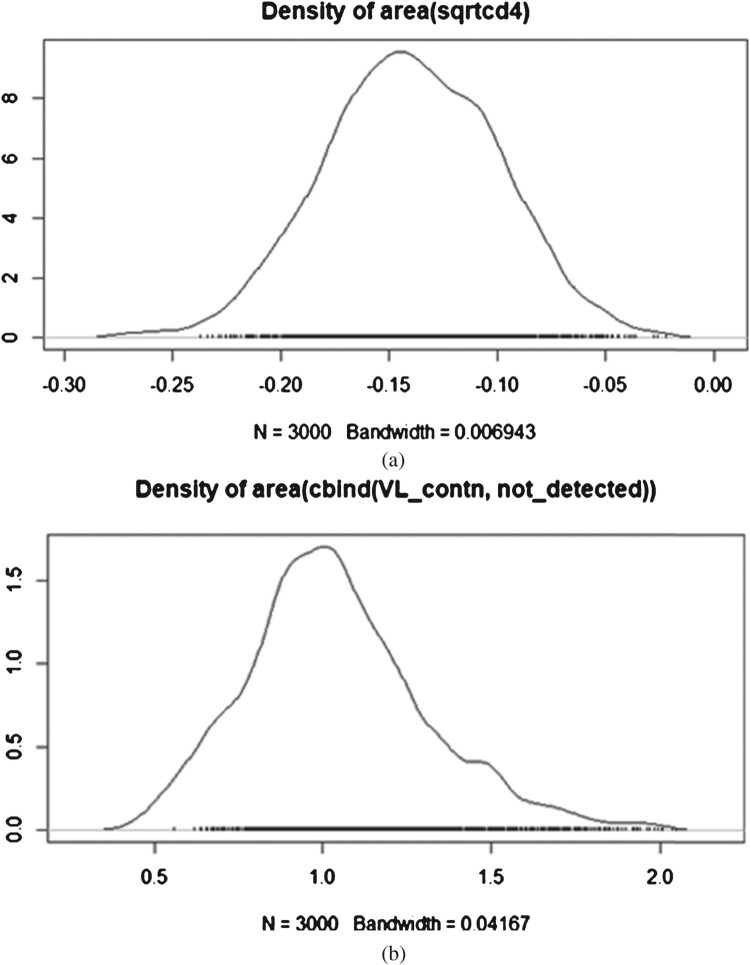
Kernel density plots for the area under the longitudinal profiles of log viral load and square root CD4 count with three chains of 3000 iterations. (a) Kernel density plot for CD4 count (b) Kernel density plot for viral load.

Overall, the chosen joint model with the area functional form did not produce any diagnostic concerns. All parameters appear to have converged to their posterior distributions, as evidenced by a high degree of agreement between the Markov chains. Specifically, the trace plots depicted in Figure 5 for the area under the longitudinal profiles of log viral load and square root CD4 count revealed that the chains overlapped consistently and sampled from the same range of values over the course of the post-burn-in iterations for all parameters regardless of the viral showing some separation at the burn-in period. In addition, the density plot depicted in Figure 6 for the parameter ‘alphas’ demonstrates a unimodal distribution for the area under the longitudinal profiles of log viral load and square root CD4 count, indicating that the parameter estimates are consistently cantered around a single most common value. The smooth shape of the density plot suggests that the variability and distribution of the estimates follow a consistent pattern. Overall, these results confirm the robustness and reliability of the parameter estimates in our joint model.

### 7.4. Personalized screening intervals for CD4 count and viral load

**Figure 7.**
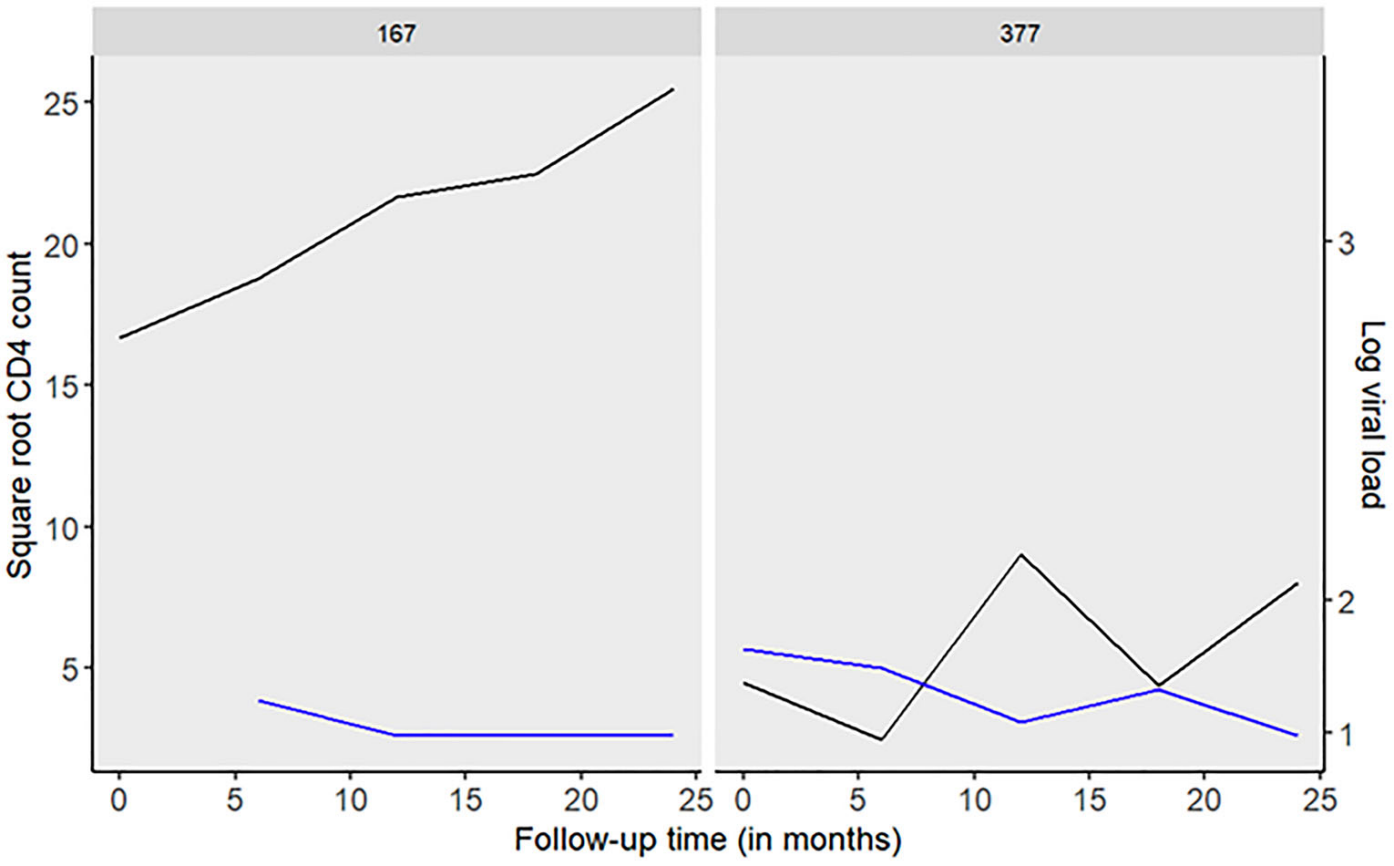
Observed longitudinal profiles for square root CD4 count (cells/mm^3^) and log_10_ viral load for two randomly selected patients. A low CD4 count (e.g CD4 < 100) a high viral load (e.g >100,000 copies/ml) indicates a worsening of a patient's condition.

To showcase the application of our methodology for determining the optimal timing of the next screening visit, we focus on CD4 count and viral load measurements as pivotal biomarkers for HIV disease progression. We consider two random patients from the SAPIT dataset: Patient 167, a 34-year-old black male, and Patient 377, a 27-year-old black male, both still alive. The longitudinal trajectories of these patients are depicted in Figure 7. Patient 167 demonstrates an increasing trajectory of CD4 count (median CD4 count: 505; IQR: 469–650) and a stable viral load (median: 400; IQR: 400–400), suggesting relative stability during the follow-up period. In contrast, Patient 377 displays a decreasing CD4 count profile (median CD4 count: 20; IQR: 19–64) and an elevated viral load (median: 16600; IQR: 1200–101000), indicating a decline in health. Table 5 presents the results for personalized screening intervals for these two patients from univariate joint models (CD4 count and viral load separately) and multivariate joint model (CD4 count and viral load simultaneously) at 2% and 5% risk thresholds. All calculations are based on the joint model with the area parameterization. For Patient 167, who maintained relative stability during the follow-up period, the results from the multivariate joint model at a 5% risk threshold suggest waiting and returning for the next measurement after 10.3 months, deviating from the conventional 6-month interval protocol. Additionally, the method recommends subsequent measurements every 12 months instead of the standard 6-month intervals (Table 5). Conversely, for Patient 377, who presented lower CD4 count levels and a high viral load, our method advises a return after 3.5 months instead of the usual 6-month wait before the next measurement. Instead of waiting for 12 months, the method suggests returning after 6.2 months and subsequently every 12 months (Table 5).

**Table 5.** Personalized screening intervals at 2% and 5% thresholds for the three models for two patients from our data.

	CD4 count		Viral load		CD4 & viral load	
Screening intervals (in months)	2%	5%	2%	5%	2%	5%
*Patient: 377*						
Visit 1	1.2	2.3	1.2	3.5	1.2	3.5
Visit 2	6.2	6.2	6.2	7.9	6.2	6.2
Visit 3	11.3	12.0	12.0	12.0	11.3	12.0
Visit 4	12.0	12.0	12.0	12.0	12.0	12.0
Visit 5	12.0	12.0	12.0	12.0	12.0	12.0
*Patient: 167*						
Visit 1	4.6	9.2	6.9	7.7	6.9	10.3
Visit 2	9.4	12.0	12.0	12.0	12.0	12.0
Visit 3	12.0	12.0	12.0	12.0	12.0	12.0
Visit 4	12.0	12.0	12.0	12.0	12.0	12.0
Visit 5	12.0	12.0	12.0	12.0	12.0	12.0

**Figure 8.**
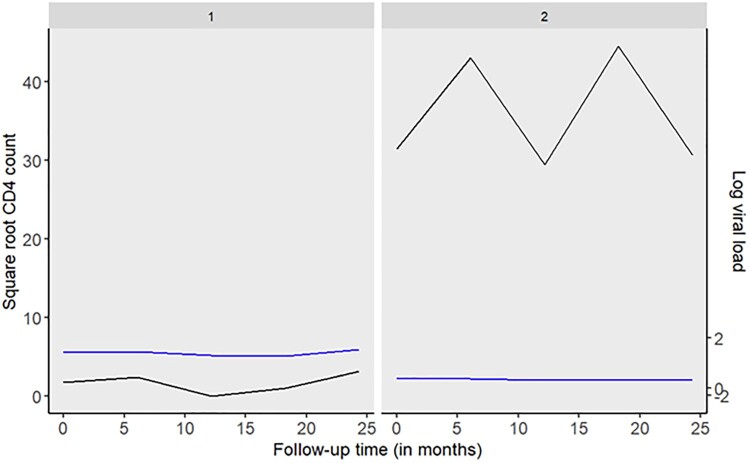
Observed longitudinal profiles for square root CD4 count (cells/mm^3^) and log_10_ viral load for two hypothetical patients. A low CD4 count (e.g CD4 < 100) a high viral load (e.g >100,000 copies/ml) indicates a worsening of a patient's condition.

To validate our results, we generated data for three patients. The longitudinal trajectories of these patients are shown in Figure 8. In particular, patient 2 exhibits very high levels of the CD4 count and low viral loads indicating a relatively stable condition throughout the follow-up period. Conversely, patient 1 exhibits a declining CD4 count profile and elevated viral load, suggesting a deterioration in health. We also simulated data for a patient 3 who experienced rapid fluctuations in their CD4 count and viral load trajectories. In particular, we simulated CD4 counts that start very low, increase, decrease, and increase again over time, and viral loads that start very high, decrease, increase, and decrease again over time. We also added random noise to both CD4 counts and viral loads to capture natural variability. These changes ensure the generated trajectories more accurately represent the complex patterns observed in clinical practice. Table 6 presents the results for personalized screening intervals for these simulated patients. Notably, the results closely mirror those obtained for actual patients in Table 5.

**Table 6.** Personalized screening intervals at 2% and 5% thresholds for the three models for three hypothetical patients.

	CD4 count		Viral load		CD4 & viral load	
Screening intervals (in months)	2%	5%	2%	5%	2%	5%
*Patient: 1*						
Visit 1	1.2	2.3	2.3	5.6	2.3	3.5
Visit 2	6.0	6.8	6.8	8.5	6.0	6.8
Visit 3	12.0	12.0	12.0	12.0	12.0	12.0
Visit 4	12.0	12.0	12.0	12.0	12.0	12.0
Visit 5	12.0	12.0	12.0	12.0	12.0	12.0
*Patient: 2*						
Visit 1	12.0	12.0	5.6	12.0	12.0	12.0
Visit 2	12.0	12.0	12.0	12.0	12.0	12.0
Visit 3	12.0	12.0	12.0	12.0	12.0	12.0
Visit 4	12.0	12.0	12.0	12.0	12.0	12.0
Visit 5	12.0	12.0	12.0	12.0	12.0	12.0
*Patient: 3*						
Visit 1	2.3	5.8	4.6	8.1	4.6	8.1
Visit 2	7.7	11.9	6.8	8.5	6.8	9.4
Visit 3	12.0	12.0	11.9	12.0	12.0	12.0
Visit 4	12.0	12.0	12.0	12.0	12.0	12.0
Visit 5	12.0	12.0	12.0	12.0	12.0	12.0

**Figure 9.**
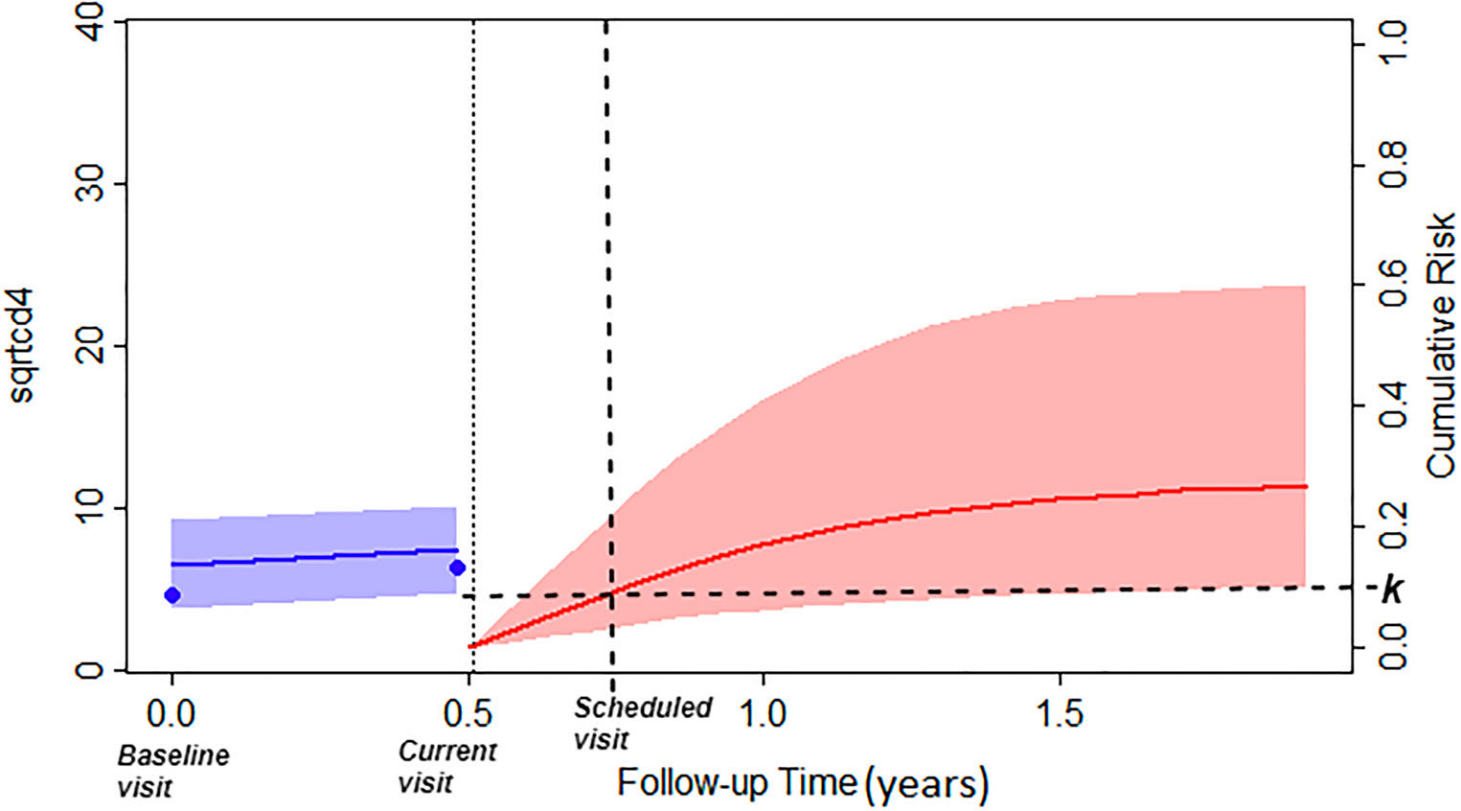
Depiction of a patient's longitudinal profile until time *t*, along with personalized decisions regarding tests influenced by the patient's individual cumulative risk of progression.

Figure 9 illustrates the methodology for screening intervals as per Table 5 for patient 377. In particular, the red line denotes the cumulative risk, and the horizontal dashed line represents the threshold *k*, and as outlined by the decision rule in Equation (2), if the cumulative risk exceeds the defined threshold, then we recommend scheduling the next visit as shown in Figure 9.

## 8. Discussion

In this paper, our objectives were twofold: first, to select the optimal model for predicting future events in patients who remained event-free, and second, to establish personalized screening intervals for CD4 counts and viral loads based on the selected model. To achieve these goals, we employed the super learning approach, estimating the integrated Brier score and the Expected Predictive Cross-Entropy (EPCE) through the combined predictions of five joint models. This approach facilitated the identification of an optimal model characterized by minimal error and high discriminatory capacity, validated through cross-validation. Our findings revealed that joint model, which has an area functional form for both CD4 count and viral load, outperformed others by exhibiting lower scores for both the integrated Brier score and EPCE, and it had the highest IBS model weights. Our findings based on this model suggested lower CD4 count measurements as well as higher viral load measurements were both strongly associated with a risk a death, emphasizing the importance of monitoring both biomarkers in HIV disease progression.

Addressing our second objective, we contributed to the literature on personalized schedules in the context of HIV by introducing innovative methodology that employs a risk-based approach to derive personalized screening intervals for CD4 count and viral load using multivariate joint models. This approach integrates comprehensive patient data, including baseline covariates, longitudinal outcomes, and previous test results, offering enhanced tailoring compared to approaches relying solely on baseline covariates and the last available longitudinal measurement (Markov assumption). Our methodology provides flexibility, allowing dynamic adjustments in screening intervals based on the evolving health status of the patient. For instance, significant fluctuations in CD4 count or viral load prompt more frequent tests, while stable health conditions lead to extended intervals, minimizing unnecessary tests. By incorporating CD4 count and viral load data into personalized risk assessments, healthcare providers can effectively tailor screening intervals for HIV patients, optimizing resources and enhancing the patient experience. This approach not only minimizes unnecessary medical procedures but also ensures timely interventions when needed. The significance of monitoring CD4 counts and viral loads as critical indicators of HIV progression is underscored by the gravity of the illness in individuals who succumbed to mortality, emphasizing the importance of timely and tailored interventions for those facing accelerated disease advancement.

Our research methodology has broader applicability beyond this specific case study. The use of extended joint models for personalized screening intervals can be applied to various biomarker studies across different disease populations, especially those involving biomarkers suffering limits of detection issues. For example, in cancer research, monitoring biomarkers such as tumor markers (e.g. PSA for prostate cancer) where assays have detection limits; in cardiovascular diseases, tracking biomarkers like troponins or NT-proBNP, crucial for diagnosing and managing heart conditions but often having detection limits; in chronic kidney disease, observing kidney function biomarkers such as creatinine or cystatin C, which may be subject to censoring; in diabetes management, monitoring blood glucose levels and HbA1c, where lower detection limits are relevant; and in infectious diseases beyond HIV/TB, applying to diseases like hepatitis, where viral load measurements are subject to limits of detection.

The TB/HIV co-infection context serves as a practical demonstration of the methodology's effectiveness in a real-world clinical setting. However, the principles underlying our approach, including multivariate joint models and personalized screening techniques, are generalizable to other disease contexts where longitudinal biomarkers are monitored.

## 9. Conclusion

While the personalized healthcare and screening approach holds substantial promise, especially in customizing interventions, its current implementation in resource-limited settings like South Africa encounters challenges rooted in constrained healthcare infrastructure, limited access to specialized diagnostic tools, data quality concerns, and financial limitations. Patient data availability and accuracy may be compromised, potentially leading to disparities in healthcare delivery based on technological access and economic status. Lower health literacy and a shortage of skilled healthcare professionals further impede effective patient engagement in personalized interventions. The cost-intensive nature of personalized healthcare, along with uncertainties about long-term sustainability, requires a careful balance between potential benefits and practical constraints. Although the current landscape may pose challenges for South Africa, the methodologies and results presented in this paper could serve as valuable guidance for future clinical trials based on biomarker-guided therapy for the effective implementations of personalized approaches. While the benefits of this approach in the context of HIV/TB screening intervals and targets remain unexplored, our results offer insights that could significantly contribute to enhancing prevention of adverse clinical events in the future.

## 10. Areas of future work

Future work could enhance Super Learning techniques, integrate advanced machine learning for model selection, and explore alternative evaluation metrics. Incorporating external data sources, such as electronic health records and genetic data, may improve insights into disease progression and risk of death. Validation with independent datasets will also be essential to assess model generalizability. Furthermore, implementing practical personalized screening interventions in clinical settings and evaluating their cost-effectiveness could guide healthcare decision-making. In the SAPiT dataset, 6.9% of baseline viral load measurements were missing, assumed to follow a missing at random (MAR) mechanism. Joint models effectively handle missing data by integrating random effects and longitudinal sub-models, ensuring robust parameter estimation. Future work will include sensitivity analysis to test deviations from the MAR assumption, using pattern-mixture based multiple imputation [38,49] to account for missing data mechanisms and unobserved variables.

## Data Availability

The data that support the findings of this study are available from CAPRISA but restrictions apply to the availability of these data, which were used under license for the current study, and so are not publicly available. Data are however available from Professor Kogieleum Naidoo who is a principal investigator of the SAPIT study (Kogie.naidoo@caprisa.org).
